# Reinhardt Rüdel: Muscle physiologist, mentor, role model

**DOI:** 10.1007/s00424-023-02886-w

**Published:** 2023-11-28

**Authors:** Christoph Fahlke

**Affiliations:** Institute of Biological Information Processing, Molekular- und Zellphysiologie (IBI-1), Forschungszentrum Jülich, 52425 Jülich, Germany

Reinhardt Rüdel, the former Chair of the Department of General Physiology at the University of Ulm, died on January 4, 2023, aged 85 years. He was a man of many interests and talents, who excelled in many positions and roles during his life, and left his mark on many of us.

Reinhardt was born on July 6, 1937, in Hochstadt in Oberfranken, Germany. After studying physics, he earned his diploma and later his PhD at the Max - Planck - Institute for Nuclear Physics in Heidelberg. After his thesis defense, Reinhardt changed his discipline to Physiology and joined the laboratory of Wolfgang Trautwein at the University of Heidelberg as a postdoc. Here, he studied cardiac electrophysiology in close collaboration with Josef Dudel and Klaus Peper, and electrophysiology remained a key technique in his laboratories throughout his career. After 3 years in Heidelberg, Reinhardt moved to University College London to work in Sir Andrew Huxley’s laboratory. A period abroad was crucial to Reinhardt’s concept of a scientific career, and he had already discussed his intention of working in London in his first negotiations with Trautwein. Reinhardt greatly enjoyed his time in London and was proud to have worked with Huxley: I remember him telling me several times that “one should work once with a genius.” In Huxley’s lab, he identified himself as a muscle physiologist, a topic he studied at multiple scales during his research career. His work on muscle evolved through a close and fruitful collaboration with Stuart Taylor, which lasted for many decades and led to one of his most important scientific contributions: the analysis of Ca^2+^ transients in contracting intact muscle fibers [1]. For these experiments, Reinhardt and Stuart used aequorin, a calcium-activated photoprotein from *Aequorea victoria*; thus, they were among of the first physiologists to utilize fluorescence measurements to follow real-time events in living cells. After his return to Germany, Reinhardt completed his habilitation thesis in Physiology at the University of Heidelberg in 1970, and in 1972 followed Josef Dudel from Heidelberg to Munich, where he remained as Professor until 1979. In Munich, Reinhardt started to work on muscle diseases, the topic closest to his heart and for which he is best known.

After first studying the classic chloride channelopathy, myotonia, he next focused on other muscle disorders with a distinct disease mechanism [2, 3]. Together with Frank Lehmann-Horn and in close collaboration with Ken Ricker from the University of Würzburg, Reinhardt published groundbreaking work on episodic paralysis and paramyotonia [4–6]. When he moved to Ulm as Chair of General Physiology in 1979, the molecular and cellular pathophysiology of muscle diseases remained his central research interest. In his typically unconventional way, he went further and also contributed to improving the living conditions of affected people and their families. Reinhardt, Ken, and Frank together produced movies to make these rare diseases better known by both patients and physicians (for example, paramyotonic paralysis by coldness – elektrophysiologic study of a muscle-membrane-defect, https://av.tib.eu/media/13981). He became a member of the scientific advisory board of the German Society for People with Muscular Diseases (DGM) and was elected first to the board of the DGM and subsequently as its first chairman from 1986 to 1992. Reinhardt was one of the first to understand the importance of diseases to basic biomedical research: knowledge of the molecular and cellular physiology of diseases is necessary for finding causal treatments and can contribute significantly to understanding the normal function of the affected cells and organs.

**Fig. 1:**
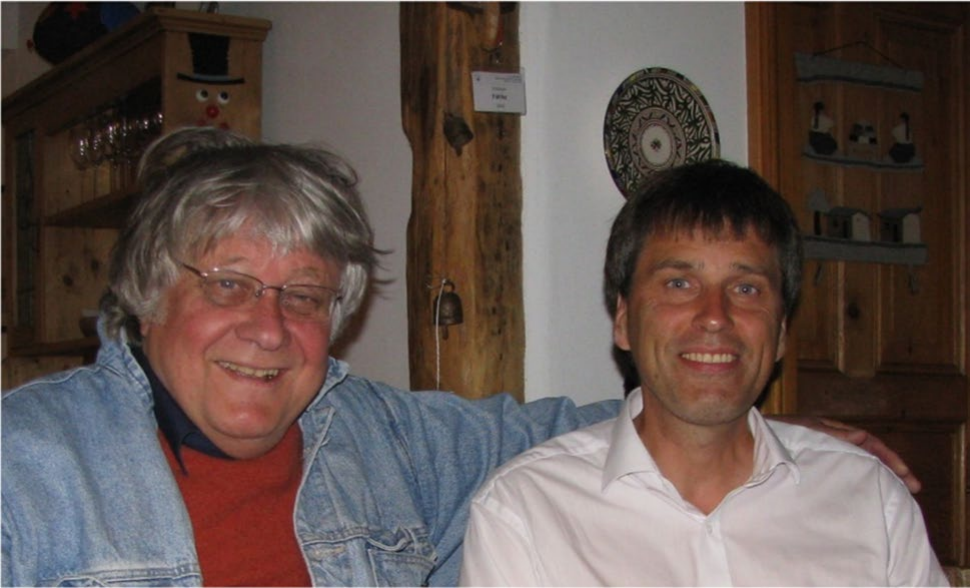
Reinhardt Rüdel and Christoph Fahlke in ChF’s kitchen in Jülich in 2014

Reinhardt was a wonderful mentor. We all enjoyed the freedom to pursue our own ideas, but were never left alone. Reinhardt was always interested not only in our work, but also in us. He helped us to organize our careers, consoled us in our defeats, and considered our work–life balance at a time when this term didn't really exist in universities. He supported us as we moved up the academic ranks and came to visit us in our own departments (Fig. 1). With Reinhardt, I fully understood the idea of the *Doktorvater* (doctoral advisor). I would not have chosen physiology as career path without him—he shaped the way I do science and lead a group. He was not only my boss, but also a good friend. I remember how happy I was when he called me up after I moved to Al George’s laboratory in Nashville, because I appreciated the gesture, but mainly because I genuinely missed talking to him.

Reinhardt often stated that a scientist’s only legacy is her/his scholars and, indeed, he has many. A few, whose time at the Department of General Physiology in Ulm overlapped with mine, are Peter Ruppersherg, the former Chair of Physiology in Tübingen; Frank Lehmann-Horn, who led the neighboring department in Ulm; Heinrich Brinkmeier, the Chair of Pathophysiology in Greifswald; and Bernd Fakler, who graduated with Reinhardt and became Chair of Physiology in Freiburg after completing his habilitation with Peter Ruppersberg.

Besides his scientific influence, he also provided an impressive example of how to live with a chronic disease. As in other parts of society, most physical disabilities remain hidden in the scientific community; therefore, role models for young chronically ill scientists are scarce. I met Reinhardt when he was already severely disabled, and he became wheelchair-bound shortly afterwards. Reinhardt completely ignored any restrictions imposed by his disability: he traveled to distant countries, climbed to mountain summits in the Andes [7], started to drive a motorcycle late in life, and danced in his wheelchair. When I was recently asked whether I ever doubted that I could run a university department with a disability, I could honestly answer that I never did—because of Reinhardt's example.

In recent years, Reinhardt’s health deteriorated, and he became mostly bedbound. Despite this, he remained active and in good spirits, starting new projects and relationships: he loved life and never gave-up. On January 4, he passed away. We will remember him as a visionary scientist, generous mentor, and good friend.
